# Supplementary material for: Exact Probability Landscapes of Stochastic Phenotype Switching in Feed-Forward Loops: Phase Diagrams of Multimodality
Source: Front Genet. 2021 Jul 8;12:645640. doi: 10.3389/fgene.2021.645640 (PMC8297706; doi:10.3389/fgene.2021.645640)
Supplement: Supplementary file 1 [file Data_Sheet_1.PDF]

Supplementary Material

1 INTEGER EQUATIONS APPROACH TO MODELING OF FEED-FORWARD LOOP

The equations governing the kinetics in the constructed feed-forward loop were developed as follows:

$$\frac{d[b]}{dt} = u_b^A[bA] - b_b^A[A][b];$$

$$\frac{d[bA]}{dt} = b_b^A[A][b] - u_b^A[bA];$$

$$\frac{d[c]}{dt} = u_c^A[cA] + b_c^B[cB] - b_c^A[A][c] - b_c^B[B][c];$$

$$\frac{d[cA]}{dt} = b_c^A[A][c] - u_c^A[cA];$$

$$\frac{d[cA]}{dt} = u_c^A[B][c] - b_c^A[cB];$$

$$\frac{d[A]}{dt} = s_A - d_A[A];$$

$$\frac{d[B]}{dt} = s_B[b] + k_1 * s_B[bA] - d_B[B];$$

$$\frac{d[C]}{dt} = s_C[c] + k_2 * s_C[cA] + k_3 * s_C[cB] - d_C[C].$$

Here $[A]$, $[B]$, $[C]$ are the concentrations of proteins A, B, and C respectfully. $[b]$, $[c]$ are the concentrations of genes b, and c, and $[bA]$, $[cA]$, $[cB]$ are the concentrations of genes b, and c in corresponding bound states bA, cA, cB.

Let the total amount of molecules of gene b in the system be n_b, and total amount of molecules of gene c in the system be correspondingly n_c. Assuming that the copy numbers of genes b and a are large, then the unique deterministic solution of the system of ordinary differential equations above are:

$$[b] = \frac{u_b^A n_b}{u_b^A + b_b^A[A]};$$

$$[bA] = \frac{n_b b_b^A[A]}{u_b^A + b_b^A[A]};$$

$$[c] = \frac{u_c^A u_c^B n_C}{u_c^A u_c^B + u_c^B b_c^A[A] + u_c^A b_c^B[B]};$$

$$[cB] = \frac{u_c^A b_c^B n_C[B]}{u_c^A u_c^B + u_c^B b_c^A[A] + u_c^A b_c^B[B]};$$

$$[cA] = \frac{u_c^B b_c^A n_C[A]}{u_c^A u_c^B + u_c^B b_c^A[A] + u_c^A b_c^B[B]}$$

$$[A] = s_A/d_A;$$

$$[B] = \frac{s_b n_b}{d_b} \cdot \frac{u_b^A + k_1 b_b^A [A]}{u_b^A + b_b^A [A]}$$

$$[C] = \frac{s_C n_C}{d_C} \cdot \frac{u_c^A u_c^B + k_2 u_c^B b_c^A [A] + k_3 u_c^A b_c^B [B]}{u_c^A u_c^B + u_c^B b_c^A [A] + u_c^A b_c^B [B]}$$

However the copy numbers of genes in the real systems is small, and usually does not exceed two copies. Thus the formulation of mass-action kinetics equation for $[b]$ and $[c]$ in a canonical way is not justified. Indeed in the case of $n_b = n_c = 1$, quantities $[b]$, $[bA]$, $[c]$, $[cA]$, and $[cB]$ are fractions in the interval $[0, 1]$ for ODEs, whereas they can only be equal to 0 and 1. In this case, one can compute six integer steady state solutions with respect to $[b]$, $[bA]$, $[c]$, $[cA]$, and $[cB]$ concentrations. Every each of these solutions corresponds to particular combination of gene copy numbers. They are 8-tuples $([A], [B], [C], [b], [bA], [c], [cA])$, such as

$$(s_A/d_A, s_B/d_B, s_C/d_C, 1, 0, 1, 0, 0),$$

$$(s_A/d_A, k_1 s_B/d_B, s_C/d_C, 0, 1, 1, 0, 0),$$
$$(s_A/d_A, s_B/d_B, k_2 s_C/d_C, 1, 0, 0, 1, 0),$$
$$(s_A/d_A, k_1 s_B/d_B, k_2 s_C/d_C, 0, 1, 0, 1, 0),$$
$$(s_A/d_A, s_B/d_B, k_3 s_C/d_C, 1, 0, 0, 0, 1),$$
$$(s_A/d_A, k_1 s_B/d_B, k_3 s_C/d_C, 0, 1, 0, 0, 1).$$

From this deterministic assumption of a discrete set of gene occupancies, we obtain six stable peaks for the system. However, as stated in the main text, when the rates of regulation are not well separated, these peaks are merged together. Note that this approach is different from boolean modeling, where the analysis is conducted in a qualitative way (as ON/OFF output) (Wang et al., 2012).

REFERENCES

Wang, R.-S., Saadatpour, A., and Albert, R. (2012). Boolean modeling in systems biology: an overview of methodology and applications. *Physical biology* 9, 055001
